# Insights into depression prediction, likelihood, and associations in children and adolescents: evidence from a 12-years study

**DOI:** 10.1007/s13755-025-00335-9

**Published:** 2025-02-28

**Authors:** Umme Marzia Haque, Enamul Kabir, Rasheda Khanam

**Affiliations:** 1 School of Mathematics, Physics and Computing, University of Southern Queensland, Toowoomba, Australia; 2 School of Business, University of Southern Queensland, Toowoomba, Australia

**Keywords:** Machine learning, Random forest, Support vector machine, Logistic regression, Apriori

## Abstract

**Purpose:**

The severity of depression among young Australians cannot be overstated, as it continues to have a profound impact on their mental health and general wellbeing. This study used machine learning (ML) algorithms to analyse longitudinal data, identifying key features to predict depression, assess future risk, and explore age-specific behaviours that contribute to its progression over time. The results emphasize the significance of early detection to prevent unfavourable consequences and shed light on the alterations in depressive symptoms during various stages of development.

**Methods:**

Three widely regarded ML techniques—random forest (RF), support vector machine (SVM), and logistic regression (LR)—are being applied and compared with a longitudinal data analysis. Additionally, the Apriori algorithm is being utilized to explore potential relationships between health, behaviour, and activity issues with depression among different age groups (10–17).

**Results:**

The analysis results indicate that the RF model is performing exceptionally well in diagnosing depression, with a 94% accuracy rate and weighted precision of 95% for non-depressed and 88% for depressed cases. In addition, the LR model shows promising results, achieving an 89% accuracy rate and 91% weighted precision. Moreover, insights from the Apriori algorithm underscore the significance of early detection by examining potential associations between health, behaviour, and activity problems and depression across diverse age groups.

**Conclusion:**

Combining early screening programs with the RF model and the Apriori algorithm is crucial for understanding depression and developing effective prevention strategies. Emphasizing Apriori's factors and regularly updating strategies with new information will enhance depression management and prevention.

**Supplementary Information:**

The online version contains supplementary material available at 10.1007/s13755-025-00335-9.

## Introduction

Depression poses a significant public health concern, particularly among children and adolescents, where its impact on overall well-being and quality of life can be enduring. Detecting and intervening early are vital for effective management, but the complex nature of this disorder requires a multifaceted approach.

Globally, mental health disorders, with depression at the forefront, have become a mounting concern, especially among young individuals. The World Health Organization (WHO) highlights depression as the most influential factor contributing to disability in 1 out of 7 individuals (14%) aged 10–19 [1]. In Australia, approximately one in four adolescents grapples with mental health issues, underscoring the urgency of this matter [2]. Despite the growing awareness of these issues, the challenging task of early detection hampers efforts to address the well-being of young Australians.

In response to this challenge, researchers have turned to machine learning (ML) algorithms as an emerging way for identifying mental health illnesses, including depression. These ML algorithms leverage extensive datasets to evaluate patterns and make precise predictions, even identifying individuals at risk before symptoms manifest. Table 1 encapsulates summaries of methods employed and outcomes from a prior synthesis of related literature, offering insights into the landscape of methodologies and findings in this domain.

**Table 1:** Summaries of approaches and finding from prior literature reviews [3]

Method/Classifier	Dataset	Performance Metric	Reference
Convolutional neural network	EEG signals from left and right hemispheres of the brain	99.12 and 97.66% classification accuracies for the right and left hemisphere	[4]
Smoothness, significance, and sanction (SS3) supervised learning model	User provided data over social media	55% F1 value and 42%precission	[5]
Support vector machine	EEG, EOG, chin EMG, ECG, oxygen saturation (SpO2), respiration and rectal body temperature from polysomnography data	86.51% accuracy	[6]
Logistic regression	I. Age 15 or over adult data from 58 articles of online journal II. < 250 sample size of various adverse health outcomes for the mothers III. 57,486 elderly populations from different articles	I. OR = 1.39, P = 0.15 II. Pooled prevalence of perinatal depression was 16.3% (CI = 95%; 14.7–18.2%, P < 0.001), with antenatal depression 19.7% (CI = 95%; 15.8–24.2%, P < 0.001) and postnatal depression 14.8% (CI = 95%; 13.1–16.6%, P < 0.001) III. Pooled prevalence of depression among old age was 31.74% (95% CI 27.90, 35.59)	[7–9]
Multivariate analysis	148 Canadian university students	Strong to moderate impact of several determinants on depression	[10]
Decision tree	Facebook data	73% accuracy	[11]
Linear regression analysis	268 participants	95% confidence level and a 6% margin of error	[12]
Naive Bayes	348 people of aged 20–60	85% accuracy	[13]
Random Forest	I. 153 of individuals from social media data of writings: textual spreading, time gap, and time span II. 250 individuals (18 + age) with criminal record III. Cross-sectional data of 667 different children and adolescents with adolescents where follow-up was not possible	I. Early risk detection error with depression detection 21.67% II. 90% AUC III. 95% accuracy	[14–16]

While existing studies, summarized in Table 1, have applied ML algorithms for depression diagnosis, there is a notable gap in research focused on young individuals and none of them used longitudinal data. Previous research studies have shown that, despite limited samples and inter-participant heterogeneity, previous studies have demonstrated good convenience, often utilizing diverse data sources [2–14]. Incorporating diverse data sources including social media, MRI scans, EEG, EOG, and ECG records, researchers have developed automated diagnostic tools that supplement clinical examinations. Notably, prior cross-sectional studies primarily involved mature participants, leaving a gap in understanding depression progression in different age groups. However, these studies have mostly focused on adults, lacking insights into depression progression in various age groups. Furthermore, a notable research gap exists in identifying individuals susceptible to future mental illness. To address this, this study aims to fill these gaps by using a longitudinal dataset comprising Australian children and adolescents of 6–17 years old. By observing variations in depressive symptoms during several stages of development, this study aims to offer understanding into the probability of developing depression at particular ages. The dataset incorporates recurring observations of the same individuals at biennial intervals, facilitating the implementation of prevalent ML algorithms, including Random Forest (RF), Support Vector Machine (SVM), and Logistic Regression (LR) to detect depression.

While previous research has shown the usefulness of ML algorithms in identifying significant features related to depression, there is a need for greater understanding of how early childhood behaviours influence on the later development of depression. This study aims to fill this gap by using association rule mining to explore the association between these behaviours and depression in subsequent stages of life.

Association rule mining is an immensely efficient method to identify patterns and connections within extensive datasets [4, 5]. It enables the identification of concealed associations and patterns that may not be readily apparent through other techniques. When exploring the relationship between early experiences and mental health outcomes, association rule mining can assist in identifying intriguing relationships between numerous factors that contribute to mental health issues and provide significant insights into their underlying causes.

To address these objectives, the study utilizes the Apriori algorithm, a well-established ML technique for association rule mining [6–9]. This algorithm finds wide application across diverse domains, including large-scale data processing, numerical analysis and hypothesis testing [10, 11]. In this study, it is applied to analyse symptoms across different age groups, allowing for the identification of unique factors associated with depression at various stages of development. It reveals which activities or combinations of activities are frequently associated with depression, facilitating the identification of risk factors and enabling comparative analysis between age groups. This approach enhances the understanding of how depression manifests at different ages and supports the development of age-specific intervention and prevention strategies.

The study aims to provide a comprehensive understanding of depression across different life stages. It will explore various factors, including daily activities, parenting styles, and other attributes not fully captured by RF analysis alone. By integrating these analytical approaches, the study aims to:

1. Develop and validate a comprehensive framework for understanding and predicting depression in children and adolescents of Australia.
2. Estimate the likelihood of developing depression by considering distinct attributes across various age cohorts.
3. Identify significant contributing factors such as age-specific behaviours or activity patterns that are commonly observed in individuals with depression.

This combined approach provides valuable insights into the identification and prevention of depression in this vulnerable population. The identified associated factors will aid researchers, clinicians, and policymakers in developing effective intervention strategies aimed at the prevention and early identification of depression in children and adolescents.

## Material and methods

**Fig. 1:**
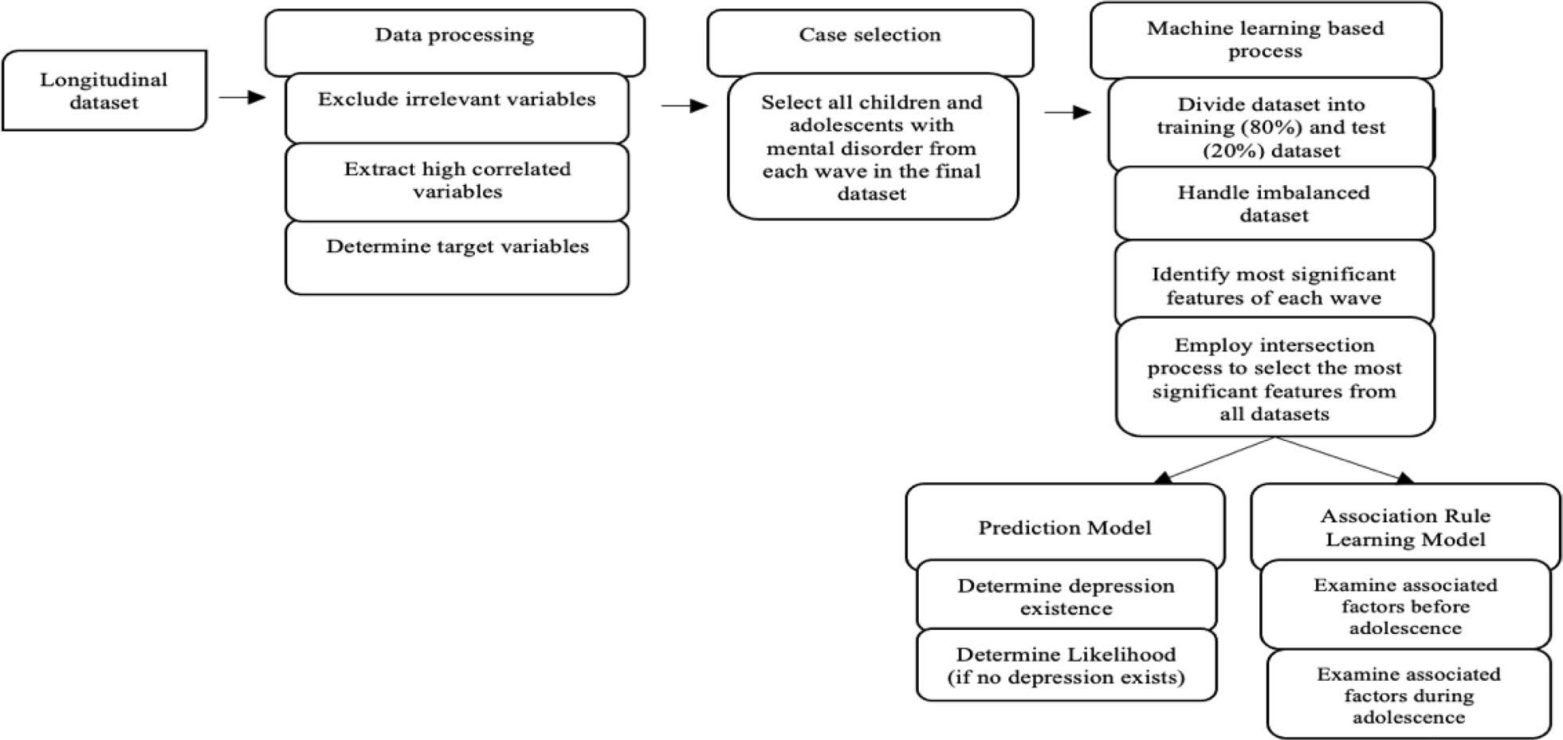
An outline of the intended framework

This study proposes a depression identification technique and examines the associations between childhood factors and depression at different ages using longitudinal data from LSAC. The proposed framework is summarized in Fig. 1.

### Dataset

The data used in this investigation has been collected using the Longitudinal Study of Australian Children (LSAC), an ongoing nationwide study has tracked the progress of a total of 10,000 children and their families within Australia since 2004. Information is obtained every two years from parents, carers, educators, and the children themselves upon reaching the suitable age bracket. The investigation has been granted ethical approval from the Longitudinal Studies Data Access Team, part of the Department of Social Services.

### Sample

The LSAC study consists of two cohorts: the birth cohort, encompassing children from birth to 1 year old, and the kindergarten cohort, consisting of children aged 4–5. Each cohort initially comprises 5000 children. The study collects a wide range of data on various aspects of the children’s well-being, social standing, and demographic factors from the beginning of childhood to early adulthood. The sampling framework for the study is based on the Medicare database managed by the Health Insurance Commission (HIC). More details about the study’s strategy and methodology are described in [12].

This research utilizes data collected using the LSAC birth cohort during the period from 2010 (wave 4) to 2020 (wave 9). Wave (4–9) represents the age group (6–7), (8–9), (10–11), (12–13), (14–15) and (16–17) respectively. While the birth cohort commences with 0 to 1 year old children, the analysis in this study commenced with 6-year-old children, reflecting the onset of mental health concerns at that age, and extends to adolescents aged 17 years. In order to identify the possibly enduring ramifications of depression and emphasise the significance and probability of early detection, this study has directed its attention towards parenting, family relationships, overall conduct, and psychological well-being of individuals between the ages of 6 and 17 years.

### Methodology

To develop the proposed approach, several experiments a number of experiments have been executed employing the Python 3.7.3 sci-kit-learn library. The methodology involves several steps, starting with data processing to eliminate irrelevant variables and identify the target variable. This step is also involved extracting the data and removing variables with lower correlation between the target variable with the other possible independent variables in the dataset. The goal is to create a final dataset that includes instances of variables from all wave’s datasets used, based on the selected case identification (id).

However, there have been challenges in this approach. One challenge is determining how to select the feature set, considering the presence of multiple datasets from different time points. Another challenge is selecting the study child, taking into account the potential changes in their mental condition across different datasets during data collection. To address these challenges, the comprehensive longitudinal dataset spanning various age groups and survey waves (4–9) is utilized in the study. Initially, the selection of key features for predicting depression has been conducted through the utilization of both RF and Boruta algorithms.

After identifying these features, the Apriori algorithm has been employed to investigate the association between health, behaviour, and activity issues and depression at across age groups ranging from 6 to 17 years, with the aim of predicting depression at the age group (16–17). This analysis aims to evaluate the broader impact of early childhood behaviours, so all relevant variables from ages (0–17) are included. Overall, this approach involves a systematic process of data processing, variable selection, case selection, ML-based prediction, and association rule mining to develop a comprehensive understanding of the factors contributing to depression among children and adolescents.

#### Data processing

The dataset has undergone a series of pre-processing steps to ensure its quality and suitability for analysis. Initially, variables such as age, area code, job code, and date of birth have been excluded from the dataset as they were deemed irrelevant or potentially violating privacy concerns. Additionally, variables with a missing data percentage exceeding 70% were eliminated, leading to the exclusion of exhibiting over 2000 instances of missing values. and rows with missing values. This meticulous approach resulted in a dataset containing 1711 variables. This methodological approach is designed to assure the integrity and reliability of the subsequent analysis, thereby facilitating effective and trustworthy findings. It aims to contribute to more accurate predictions by minimizing biases and maintaining data integrity.

#### Data extraction

The process involves considering each wave for data extraction, followed by conducting a chi-square test for the assessment of the association in between categorical variables and the outcome variable. Variables having p-values below 0.05 are advanced to the subsequent stage of analysis, indicating their association in relation to the outcome variable. Subsequently, correlation analysis is performed using various methods, including Phi coefficient, Point Biserial, and Tetrachoric correlation, given the dataset’s binary nature. Despite the diverse methods, the results remain consistent due to the binary variables’ inherent characteristics, where calculating covariance equates to correlation coefficient computation. However, Tetrachoric correlation is selected due to its suitability in situations where the underlying continuous variables are not explicitly observed but are presumed to adhere to a normal distribution [13].

#### Variable selection

Variable selection is an important step in the methodology, especially for longitudinal data analysis. After the data processing stage, the remaining relevant variables with lower missing data are retained for further analysis. This ensures that the dataset is suitable for the subsequent investigations and provided confidence in the validity of the forthcoming conclusions. The target variable is determined by this query, “Would you describe the child’s anxiety disorder and depression as nondepressed, mild, moderate, or severe?”. The response of the target variable is either classified as ‘depressed’, denoted by a value of 1 (indicating moderate to severe) or ‘non-depressed’, denoted by a value of 0 (indicating nondepressed to mild). This stage encompasses all instances of psychiatric disorder cases collected from various waves, enabling the assessment of progression of symptoms. Categorical variables undergo one-hot encoding. Subsequently, the dataset undergoes the entire process of extracting data, wherein variables exhibiting low correlation with the target variable are eliminated, yielding 165–170 variables from the total variables in each wave.

#### Case selection

The goal is to create a final dataset that includes instances of relevant variables from all datasets, based on the selected case id. Cases with mental disorders and without mental disorders are chosen from each dataset depending on the child’s psychiatric condition (‘depressed’ or ‘non-depressed’). These selected cases are then considered for instances from other waves, resulting in a dataset of 1785 individuals aged between 6 and 17 years. By combining these selected cases, this study aims to identify the most significant features associated with different levels of depression, ranging from mild to severe.

#### Handling imbalanced dataset

To identify the most significant features associated with different levels of depression, the study aims to combine these selected cases. However, the resulting dataset shows an imbalance, with the ‘non-depressed’ class accounting for 74% of the data, while the ‘depressed’ class represents only 26%. In ML, addressing class imbalance is crucial to prevent bias and ensure model performance. To rectify this imbalance in class distribution, the Synthetic Minority Oversampling Technique (SMOTE) is utilized. This technique involves creating synthetic instances for the minority class by randomly sampling data from the majority class. This technique contributes to a more balanced dataset, improving the model’s ability to accurately classify both classes [14].

#### Feature selection

To identify the most significant features among highly correlated variables within each dataset, the Boruta method alongside a RF classifier has been employed to select features in an impartial and robust manner. This technique removes irrelevant or redundant features and identifies the significant and relevant characteristics related to the target variable [15, 16].

It is important to note that that the features in these datasets exhibit nearly identical characteristics across various time points. To combine these datasets, the significant features from all of them can be added together using the union operation. However, this method might result in a larger feature space than necessary, potentially adding noise or irrelevant features. This could impact the accuracy of any further analysis or modeling performed on the combined dataset. Certain characteristics may hold diverse implications or connotations across distinct datasets, potentially influencing the reliability of any analysis or modeling performed on the integrated dataset.

To mitigate these issues, the intersection process is utilized to choose comprehensive feature sets in cases where there is variability in features observed at different time intervals. Therefore, it is concluded that no important features are missed by this process. The eliminated features are deliberately removed because they are similar to the already identified significant features. This is further supported by the outstanding results attained from the prediction model, as mentioned in the results section.

#### Classification

This study examines and outlines renowned supervised learning models expected to deliver optimum outcomes for the longitudinal dataset.

##### Random forest (RF)

Random forest is an ensemble learning method that generates numerous decision trees and then interacts their predictions to enhance accuracy as well as stability. It builds decision trees using bootstrapped samples and randomly selects features for each split [17]. It calculates feature relevance as the average across all trees, mitigating overfitting by predicting random subsets of features and instances. This calculation involves dividing the aggregate count of trees by the combined sum of feature importance scores across all individual trees:

1 $$RFfi_{i} = \frac{{ \sum_{j \in all\,trees } normfi_{j} }}{T}.$$

where RFfi_i_ = the feature importance, i calculated from all trees in the RF model, normfi_j_ = the normalized feature importance for i in tree j and T = total number of trees.

##### Support vector machine (SVM)

SVM detects hyperplanes in multi-dimensional space to separate data into different classes, maximizing the margin between them [18]. It excels in datasets with high-dimensional feature spaces and in cases where the quantity of features surpasses the number of instances. It is equally proficient in addressing linear as well as non-linear classification challenges, employing various kernels. The equation employed to calculate probabilities for input parameters corresponding to each class is as follows:

2 $$f\left( x \right) = \left( {\mathop \sum \limits_{j = 1}^{m} \left( {a_{j} x_{ij} + a_{0} } \right)} \right)y_{j}$$

where n = number of data points, m = number of attributes, x_ij_ = ith attribute of jth data point, a_j_, a_0_ = the model parameters (weights and bias), and y_j_ = the class label of the j-th data point (+ 1 or −1).

##### Logistic regression (LR)

LR estimates the likelihood of a categorical outcome variable determined by considering predictor variables, differing from linear regression’s prediction of continuous responses. It performs effectively in linear classification scenarios, demonstrating proficiency in both binary and multi-class categorization. The logistic function (sigmoid function) calculates the outcome probability using the given equation [19]:

3 $$\hat{p} = \frac{{e^{{\left( {b_{0} + b_{1} X} \right)}} }}{{1 + e^{{\left( {b_{0} + b_{1} X} \right)}} }}$$

where n = number of data points, m = number of attributes, xij = ith attribute of jth data point, aj, a0 = the model parameters (weights and bias), and yj = the class label of the j-th data point (+ 1 or − 1).

##### Performance metrics

Evaluation of the ML algorithms involved analyzing True Positive (TP), True Negative (TN), False Positive (FP), and False Negative (FN) outcomes via a confusion matrix. The accuracy, precision, recall, and F1 scores for each model have been computed using the provided equations:

4 $${\text{Accuracy Rate}} = \frac{TP + TN}{{TP + FP + TN + FN}}$$

5 $${\text{Precision}} = \frac{TP}{{TP + FP}}$$

6 $${\text{Recall}} = \frac{TP}{{TP + FN}}$$

7 $${\text{F1 score}} = \frac{2*Precision*Recall}{{Precision + Recall}}$$

##### Area under receiver operating characteristic curve (AUC) score

The AUC score provides a comprehensive assessment of the classifier’s performance by comparing the true positive rate (sensitivity) with the false positive rate, ranging between 0 and 1. Higher values signify superior ability of the model to detect positive cases [19].

After employing ML supervised models to detect depression among children and adolescents, the aim of this research is to explore the association between health, behaviour as well as activity issues at ages 6 to 17 with consequent, depression at age group (16–17). To achieve this, an association rule mining algorithm called Apriori is employed to identify the variables that often appear together in relation to depression at age group (16–17).

#### Association rule mining

In addition to predicting depression, this study seeks to examine the link between health, behaviour, and activity issues and depression in children and adolescents of different age groups. To achieve this, this study has employed the Apriori algorithm, a well-established technique renowned for its effectiveness in association rule mining [20]. The aim is to uncover potential risk factors that may be contributed to the development of depression later in life. By utilizing the Apriori algorithm, specific patterns of symptoms that are commonly occurring in individuals with depression at different ages are detected. This enhances our understanding of the distinct factors associated with depression at various stages of life. The Apriori algorithm, along with the FP-Growth algorithm, are widely used in mining techniques for associative rules. Through this process, the study has identified a group of frequently observed influential factors. Each variable within this group serves as the determining factor (antecedent) for the frequently occurring variables (consequent) in the Apriori algorithm.

##### Apriori

The Apriori algorithm, a fundamental technique in association rule mining, follows a series of steps for rule generation [21]. It begins with identifying frequently occurring itemset, represented as (X, Y), where X and Y are variables forming an association rule. Subsequently, significant patterns such as (X → Y) are discovered, indicating the presence of Y in all items containing X. The algorithm applies the Apriori principle to construct subsets of commonly appearing variables, which are then refined using the Apriori algorithm. The resulting association rules are selected based on measures like support, confidence, lift, and conviction. Finally, the algorithm identifies maximal frequent itemset and closed frequent itemset to uncover associations and patterns in large datasets.

##### Performance measure

To assess the effectiveness of the approach, four key metrics are computed: support, confidence, lift, and conviction.

Support:

Support indicates how often an item appears in the dataset. The support for the combination of X and Y is determined by the following equation:

8 $${\text{Support}}({\text{X}} \to {\text{Y}}) = \frac{{\text{transactions that contain both X and Y }}}{{\text{Total Transactions}}}$$

Confidence:

Confidence gauges the trustworthiness of a rule, representing the likelihood of the consequent (Y) based on the antecedent (X). The confidence measurement is expressed as:

9 $${\text{Confidence}}\left( {{\text{X}} \to Y} \right){ } = { }\frac{{\text{transactions that contain both X and Y }}}{{\text{Transactions containing Y}}}$$

Lift:

Lift measures the strength of the relationship between the elements of a rule. The calculation is performed using the following equation:

10 $${\text{Lift}}\left( {{\text{X}} \to Y} \right){ } = { }\frac{{{\text{Support}}\left( {{\text{ X}} \to {\text{Y }}} \right)}}{{{\text{Support}}\left( {\text{X}} \right){\text{*Support}}\left( {\text{Y}} \right)}}$$

Conviction:

Conviction assesses the likelihood of one event happening without being influenced by another event, even if they are interdependent. The conviction measurement is given by:

11 $${\text{Conviction}}\left( {{\text{X}} \to Y} \right){ } = \frac{{1 - {\text{ Support}}\left( {\text{Y}} \right)}}{{1 - {\text{Confidence}}\left( {{\text{X}} \to {\text{Y}}} \right)}}$$

The combined value of these metrics provides an extensive evaluation of the method’s performance by considering aspects such as frequency, reliability, strength of association, and conditional probability.

## Results

This section comprises two parts: the predictive model and the association rule mining model. The first part aims to identify representative features for the predictive model. Subsequently, the section provides an overview of how the models perform on the testing dataset in terms of classification. It illustrates how the best-fitting model utilizes essential input attributes to generate decision outcomes for typical test scenarios. Finally, the section concludes by presenting the results of association rule mining, specifically Apriori analysis, which explores the relationship between early childhood factors and depression at different ages.

### Prediction model

In the predictive model, the dataset is partitioned into an 80% training dataset and a 20% test dataset. The most significant features are then determined using Boruta on RF to recognize the most impactful variables. Table 2 provides a succinct summary of these significant features identified in the study.

**Table 2:** Most significant features [3]

Identified sign/symptoms of depression	Description of variables
Medical condition: Nervousness^a,b^	Does the study child have nervous condition?
Coping^a,b^	Does the study child have a difficulty or delay in any of the following areas compared to children of a similar age? Cope with emotions
Social and emotional outcomes: Reacts strongly to disappointment^a,b^	Does the study child react strongly (cries or complains loudly) to a disappointment or failure?
Homework incomplete unless reminded^a,b^	Does the study not complete homework unless reminders are given?
Difficulty completing assignments^a,b^	Has difficulty completing assignments (homework, chores.)?
Complained of headaches etc.^a^	Does the study child complain of headaches etc.?
Often seemed worried^a,b^	Does the study child often seem worried?
Often been unhappy or tearful^a^	Does the study child often seem unhappy?
Easily lose confidence^a^	Does the study child often lose confidence?
Had many fears^a^	Does the study child have many fears?
Temperament^a,b^	Does the study child become angry frequently?
Emotional development: Problems feeling afraid or scared^a^	Has the study child has had a problem with this?
Problems feeling sad^a^	Has the study child has had a problem with this?
Trouble sleeping^a^	Has the study child has had a problem with sleeping?
Social development: Unable to do what other children can^a,b^	Has the study child has had a problem with this?
Problems keeping up with other children^a,b^	Has the study child has had a problem with this?
School readiness: Problems missing days due to illness^a^	Has the study child has had a problem with this?
Parental involvement: Contacted school about attendance^a^	Has the parent contacted the school for various reason?
Parent living elsewhere: Study Child excitement on arrival in home^a^	Does the study child become excited on the parent’s arrival?
Social development Helpful if someone is hurt etc.^a,b^	Is the study child helpful if someone gets hurt?

^1^Asked to parent (father/mother)

^2^Asked to teacher

The dataset includes data from 1785 children and adolescents, representing 20% (n = 357) of each wave. Among these individuals, 26% (n = 464) are identified as individuals affected by depression across all waves. Table 3 presents the reports regarding classification along with the assessment metrics for the depression detection models, including RF, SVM with linear kernel, LR.

**Table 3:** Depression detection with ML models [3]

RF classification report									
	Precision		Recall		F1-score			Support	
Negative	0.96		0.95		0.96			261	
Positive	0.88		0.89		0.88			96	
Accuracy					0.94			357	
Macro avg	0.92		0.92		0.92			357	
Weighted avg	0.94		0.94		0.94			357	
Accuracy: 94%									
Weighted precision: 95%									

SVM classification report									
	Precision		Recall			F1-score		Support	
Negative	0.93		0.95			0.94		261	
Positive	0.84		0.79			0.82		96	
Accuracy						0.90		357	
Macro avg	0.88		0.87			0.88		357	
Weighted avg	0.90		0.90			0.90		357	
Accuracy: 90%									
Weighted precision: 95%									

LR classification report									
		Precision		Recall			F1-score		Support
Negative		0.91		0.92			0.92		261
Positive		0.78		0.76			0.77		96
Accuracy							0.88		357
Macro avg		0.84		0.84			0.84		357
Weighted avg		0.88		0.88			0.88		357
Accuracy: 88%									
Weighted precision: 91%									

**Fig. 2:**
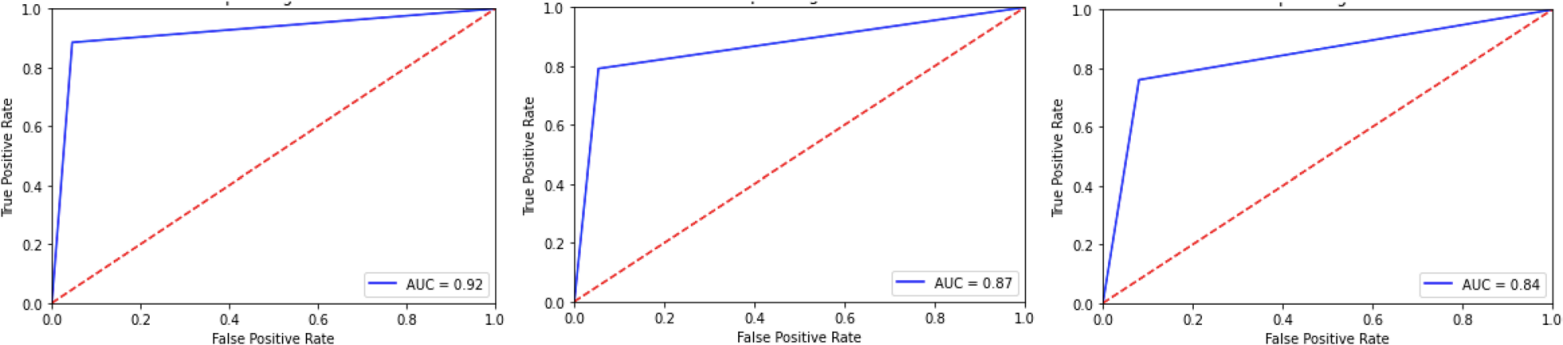
AUC scores for the **a** RF, **b** SVM and **c** LR models [3]

Table 3 presents the efficacy of the proposed system in ML-based classification. Among classifiers utilized, RF-based classification outperforms the other classifiers, achieving the highest accuracy of 94% across various metrics. SVM follows closely with an accuracy of 90%, whereas the LR-based method trails behind with an accuracy of 88%. Additionally, the performance evaluation includes the assessment of AUC scores for these classifiers, illustrated in Fig. 2. RF attains the highest AUC score of 92%, denoting its exceptional discriminatory capability. SVM and LR follow with AUC scores of 87 and 84%, respectively [3].

**Fig. 3:**
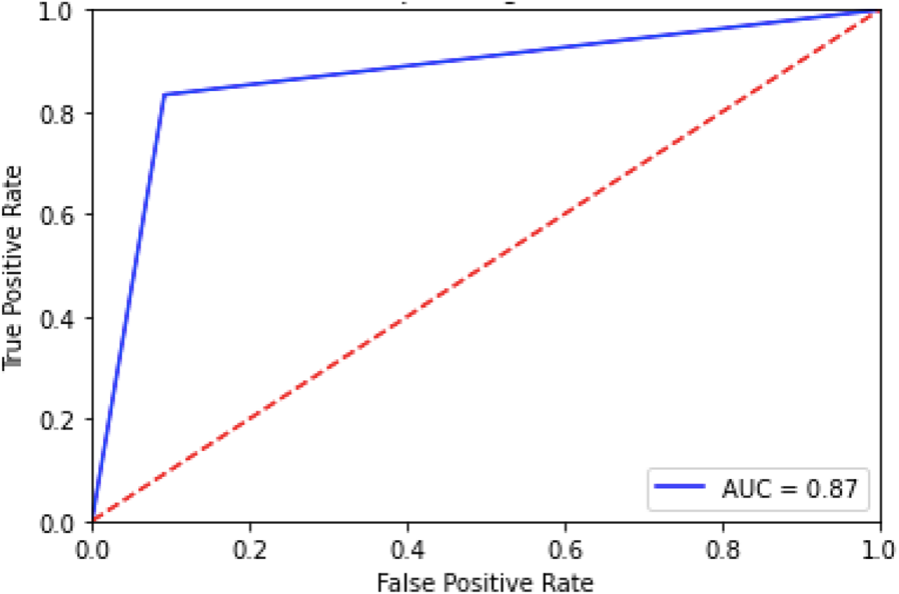
AUC score in assessing depression likelihood using LR [3]

Moreover, the study explores the likelihood of experiencing depression across diverse age groups (6–17). LR is utilized to evaluate the probability of depression occurring during later adolescence, aged between (10–17) based on significant features observed during earlier ages (6–9). Table 4 shows the LR outcomes regarding depression likelihood, revealing an accuracy rate of 89% [3]. Additionally, Fig. 3 illustrates the AUC score in the depression likelihood assessment across different age categories with LR, showing a value of 87% [3].

**Table 4:** Assessment of depression likelihood using LR for classification [3]

LR Classification Report				
	Precision	Recall	F1-score	Support
Negative	0.94	0.91	0.92	261
Positive	0.77	0.83	0.80	96
Accuracy			0.89	357
Macro avg	0.85	0.87	0.86	357
Weighted avg	0.89	0.89	0.89	357
Accuracy: 89%				
Weighted precision: 91%

### Association rule mining model

Apriori analysis is employed as the association rule mining model to determine the factors associated with depression during the critical developmental stage of ages (16–17) [22]. To achieve this, all 165–170 variables obtained from the data extraction process of each wave are considered. The Apriori algorithm is employed to identify rules with high lift and conviction values, which indicate a strong relationship with depression and serve as strong predictors of depression.

It is worth highlighting that a rule with a support value less than 1 Implies a low frequency of association within the dataset. However, if both lift and conviction values surpass 1, it signifies a robust association, although the rule has minimal overall support. Higher lift and conviction values suggest a positive influence of the rule body on the presence of the rule head.

Significant factors associated with depression across various age groups (6–17), particularly those linked to depression within the age group (16–17), are identified through Apriori analyses conducted at different confidence levels. This section explores the strong factors associated with depression at different age groups that are connected to depression at age group (16–17).

#### Strong associated factors of depression before adolescence linked to depression at age group (16–17)

**Fig. 4:**
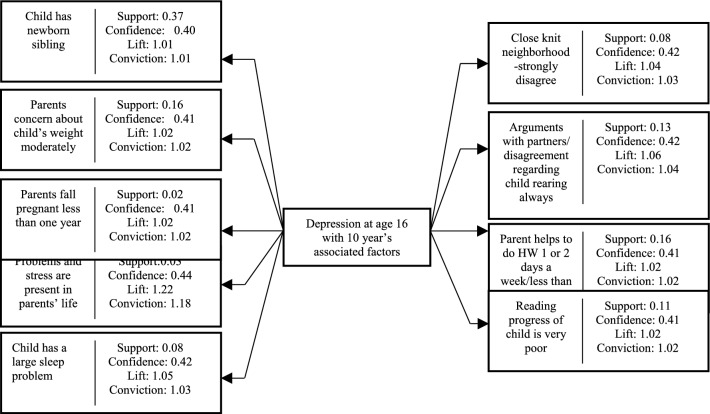
Factors linked to depression in the age (10–11) and their impact on depression in the age group (16–17)

**Fig. 5:**
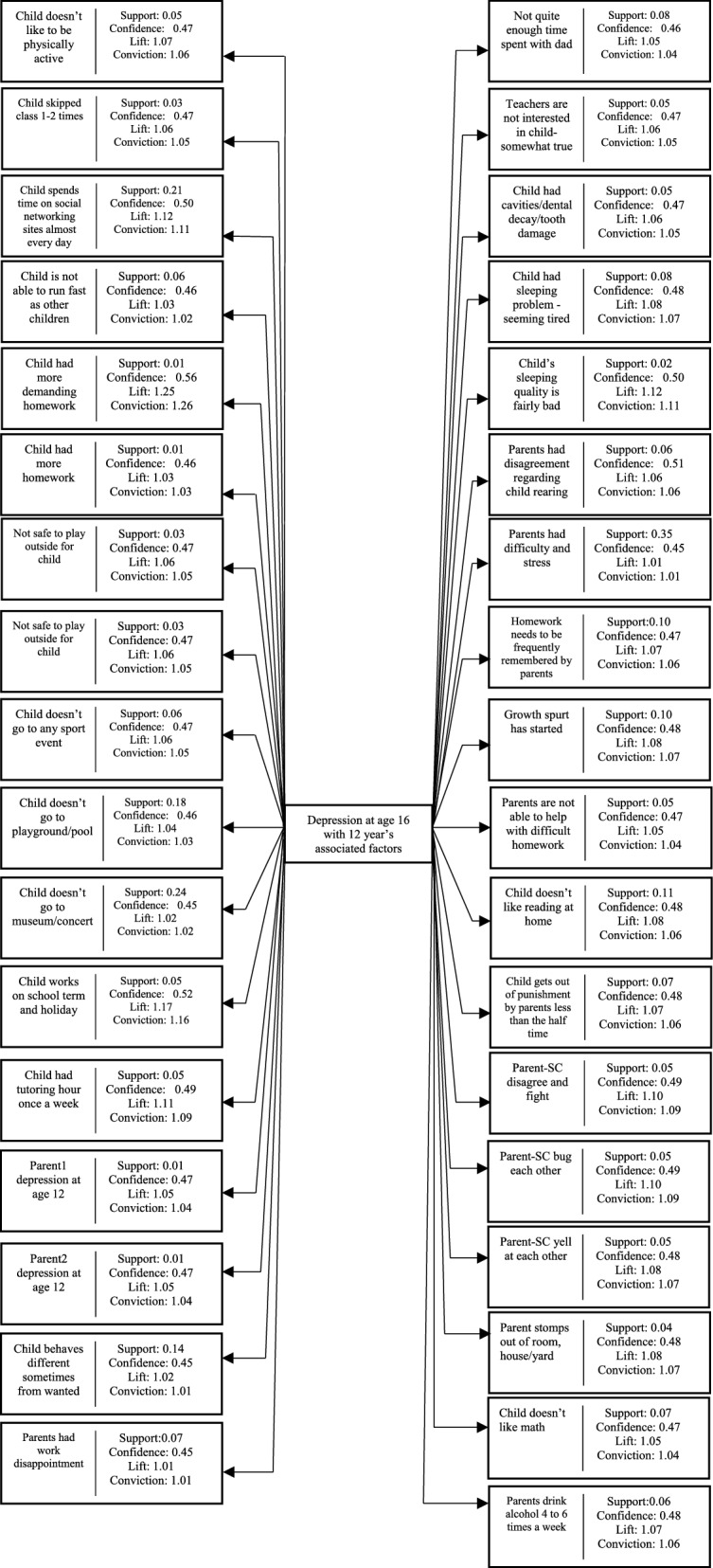
Factors linked to depression in the age (12–13) and their impact on depression in the age group (16–17)

**Fig. 6:**
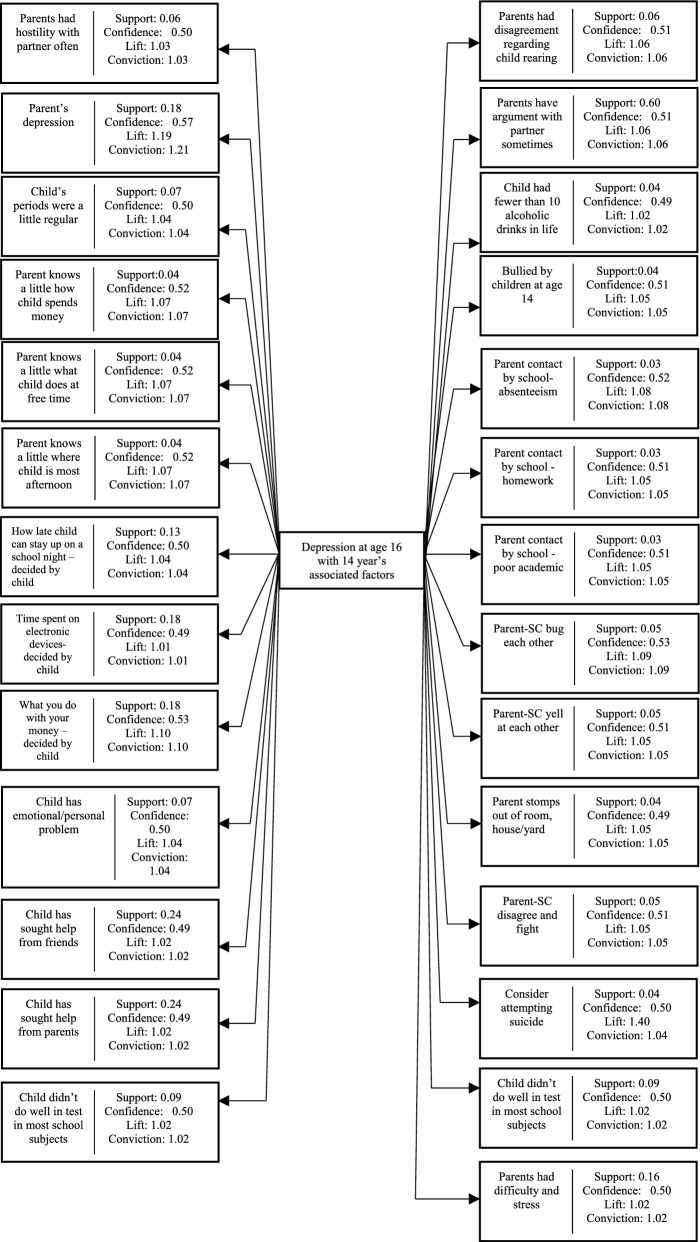
Factors linked to depression in the age (14–15) and their impact on depression in the age group (16–17)

**Fig. 7:**
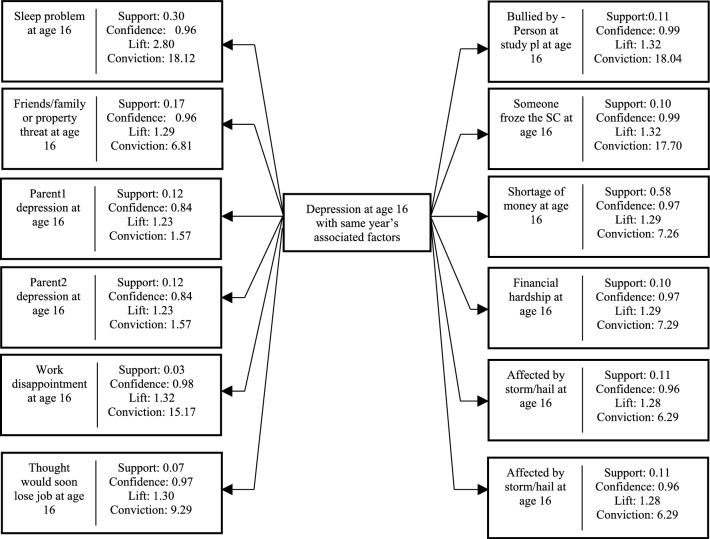
Factors linked to depression in the age (16–17) and their impact on depression in the age group (16–17)

In the age groups (6–7) and (8–9), no factors are found significantly associated to depression within the age group (16–17), as indicated by low lift and conviction values. This analysis has revealed significant factors for ages ranging from 10 to 17. The factors associated with depression within the age group (16–17) are illustrated in Figs. 4, 5, 6 and 7 in the Appendix.

#### Strong associated factors of depression during adolescence linked to depression at age group (16–17)

Within the age group (10–11), a number of factors are found to be significantly associated with depression at age 16. These include the absence of a close-knit neighbourhood (lift: 1.04, conviction: 1.03), arguments with partners (lift: 1.06, conviction: 1.04), parents’ limited ability to assist with homework (lift: 1.02, conviction: 1.02), and the child’s poor progress in reading (lift: 1.02, conviction: 1.02). Additionally, parents’ stress (lift: 1.22, conviction: 1.18) and the child’s sleeping problems (lift: 1.05, conviction: 1.03) are also linked to depression.

When examining the age group (12–13), numerous factors are identified that remain strongly associated with depression at age 16. These include disagreements between parent and child, parents’ alcohol consumption (lift: 1.07, conviction: 1.06), parents are not able to help with difficult homework (lift: 1.07, conviction: 1.06), parent child disagreement (lift: 1.10, conviction: 1.09), bug and yell at each other (lift: 1.08, conviction: 1.07), parents stomping out of the room or house yard (lift: 1.08, conviction: 1.06), the child’s disinterest in physical activities (lift: 1.07, conviction: 1.06), and parents had difficulty and stress (lift: 1.06, conviction: 1.06). Additionally, new factors such as demanding homework (lift: 1.25, conviction: 1.26), feeling unsafe while playing outside (lift: 1.06, conviction: 1.05), and not participating in sport activities (lift: 1.06, conviction: 1.05) are also linked to depression.

Upon investigating the age group of (14–15), several significant factors are found to be associated with depression in the age group (16–17). These include parents’ disagreements regarding child rearing (lift: 1.06, conviction: 1.06), being bullied by other children (lift: 1.05, conviction: 1.05), school-related issues such as school absenteeism (lift: 1.08, conviction: 1.08), poor academic performance and homework (lift: 1.05, conviction: 1.05), and parents experiencing difficulty and stress (lift: 1.19, conviction: 1.21). Factors such as parents having little knowledge of how the child spends money (lift: 1.07, conviction: 1.07), what the child does during free time (lift: 1.07, conviction: 1.07) and where the child is most often in the afternoon (lift: 1.07, conviction: 1.07) are also found to be significant.

Finally, in the age group (16–17), several strong factors are discovered to be associated with depression, including being a victim of bullying (lift: 1.32, conviction: 18.04), experiencing a freezing incident caused by someone (lift: 1.32, conviction: 17.70), facing a shortage of money (lift: 1.29, conviction: 7.26), experiencing financial hardship (lift: 1.29, conviction: 7.29), being affected by a storm (lift: 1.28, conviction: 6.29), and having sleep problems (lift: 2.80, conviction: 18.12). Other factors such as the threat posed by friends, family, or property (lift: 2.80, conviction: 18.12), parents’ depression (lift: 1.23, conviction: 1.57), work disappointment (lift: 1.32, conviction: 15.17), and the fear of job loss (lift: 1.30, conviction: 9.29) are also found to be linked to depression in the age group (16–17).

To summarize, the Apriori analysis has provided valuable insights with age-specific confidence levels, identifying key contributors to depression at ages (10–17). However, for age groups (6–7) and (8–9), no factors are identified as being linked to depression within the age group (16–17), as indicated by the low lift and conviction values.

## Discussion

The proposed method utilizing a longitudinal dataset containing Australian children and adolescents aged between 6 and 17 years, the application of an RF classifier in the proposed technique has resulted in a 94% accuracy rate in detecting depression. This indicates that the model excels in identifying the manifestation of depressive symptoms. Remarkably, the precision and recall rates achieve 96 and 95%, respectively, for identifying ‘non-depressed’ instances, whereas for ‘depressed’ cases, they reach 88 and 89%, respectively.

These results are significantly better than previous studies that relied on information sourced from social media platforms and cross-sectional datasets [3, 9, 11–14]. The precision and f1 score for the ‘depressed’ and ‘non-depressed’ classes are notably higher in this study, at 88% and 96%, respectively. This represents a substantial improvement compared to the values reported in [3] where the corresponding values were 55 and 42%.

Furthermore, the accuracy score of 94% greatly surpasses the scores of 73% found in [9], 85% [11] and 78.33% [12]. Although the accuracy score shows a modest rise to 95% [14], a detailed analysis of the specific performance metrics, including precision, recall, and f1 score, indicates that the study exhibits superior outcomes in both ‘depressed’ and ‘non-depressed’ classes. Moreover, the AUC score of 92% outperforms results reported for mentally disordered offenders [13]. Overall, the investigation showcases outstanding outcomes in detecting depression within the studied population, with notable performance metrics in precision, recall, and f1 score.

Moreover, the robustness of the model is further emphasized by the macro and weighted mean performance metrics, with precision, recall, and f1-score, each exceeding 0.9. This indicates that the model is consistently accurate across diverse age groups. Additionally, the model offers valuable insights into symptom variations across diverse age cohorts, facilitating the formulation of specific strategies for prevention and intervention. The model exhibits an 89% accuracy in assessing depression likelihood across different age groups. It demonstrates precision rates of 77% for ‘depressed’ cases and 94% for ‘non-depressed’ cases. Moreover, the model achieves recall rates of 83% for ‘depressed’ individuals and 91% for ‘non-depressed’ individuals. Additionally, the f1 score stands at 80% for ‘depressed’ cases and 92% for ‘non-depressed’ cases. These insights enrich the understanding of symptom dynamics across different age brackets, aiding in the formulation of tailored intervention approaches.

After identifying symptoms across diverse age groups, the investigation focused on factors associated with depression at different ages using the Apriori algorithm. This analysis has revealed strong factors linked to depression at each age group. In the age group (10–11), depression development is influenced by environmental factors, family dynamics, and sleeping problems. Within the age group (12–13), notable associations with depression include parent–child disagreements, school-related challenges, and physical inactivity of the child. Similarly, in the age group (14–15), family dynamics, school-related issues, and personal habits play a role in depression development, while in the age group (16–17), factors such as bullying, financial hardship, sleep problems, and family dynamics strongly contribute to depression. However, no associated factors have been identified for depression in the age groups (6–7) and (8–9), as indicated by the low lift and conviction values. This highlights the significance of exploring the relationship between various factors associated with depression, particularly during adolescence (10–17 years). The absence of associated factors for age groups (6–7) and (8–9) suggests a potential difference in the dynamics of depression risk at these early ages. Children at age groups (6–7) and (8–9) are typically in early childhood and are still developing their understanding of themselves and the world around them. They may not yet have the cognitive ability to accurately report or communicate their feelings of depression or the factors contributing to it [23–25].

By utilizing ML algorithms and examining associated factors not captured by previous feature selection techniques, this research provides a more comprehensive understanding of the multifactorial nature of depression and its interactions with early behaviours. Recognizing the presence of different associated factors at different ages suggests that the factors influencing depression may change as individuals grow older. This change could be influenced by various factors such as changes in social environments, cognitive development, hormonal changes, and individual coping mechanisms.

### Social environments

Social environments play a crucial role in mental health. During childhood and adolescence, individuals are heavily influenced by family dynamics, peer relationships, and school environments [26, 27]. As individuals transition into adulthood, factors such as work, relationships, and societal expectations become increasingly influential [28, 29].

### Cognitive development

Cognitive abilities evolve across the lifespan, impacting how individuals perceive and respond to stressors [30]. In childhood, limited cognitive capacities may affect the way individuals process and express emotions. As cognitive abilities mature during adolescence and adulthood, individuals may develop more nuanced ways of understanding and coping with challenges [31, 32].

### Hormonal changes

Hormonal fluctuations, particularly during puberty and later life stages, can influence mood and emotional well-being [33, 34]. Adolescence, marked by significant hormonal changes, is a critical period where susceptibility to risk-taking behaviour may increase [35, 36].

### Coping mechanisms

Coping mechanisms evolve from simple strategies in childhood to more complex cognitive and behavioural approaches in adolescence and adulthood [37, 38]. The ability to adaptively cope with stressors can mitigate the impact of risk factors for depression [39, 40].

Understanding how factors associated with age influence depression is crucial for developing targeted interventions and preventive measures. By focusing on age-specific factors linked to depression, tailored strategies can be devised to enhance mental well-being at different life stages. This approach also aids in identifying risk and protective factors, facilitating early detection and intervention for individuals at higher risk based on their age-related factors. Furthermore, recognizing these age-specific factors can guide future research and refine prevention and intervention strategies. The integration of RF and the Apriori algorithm enables the collection of comprehensive data on depression symptoms and contributing factors across various age groups, providing valuable insights for clinicians, researchers, and policymakers. This knowledge is essential for crafting effective treatment plans and prevention strategies tailored to different age groups affected by depression.

## Limitations of the study

Several limitations within this study must be acknowledged. Firstly, the dataset exclusively encompasses Australian children and adolescents aged (6–17), necessitating caution when extrapolating the results to broader populations or different age groups. Secondly, the cross-sectional design of the study is a limitation. While the longitudinal nature of the dataset offers valuable insights into the progression of depression symptoms and associated factors over time, establishing causality is not possible. Nevertheless, it is crucial to recognize that the outcomes from this study underscore the efficacy of the data construction template.

## Conclusion

The present study investigates the effectiveness of combining a RF classifier with the Apriori algorithm in understanding and addressing depression across various age brackets in Australian children and adolescents, ranging from 6 and 17 years old. The model demonstrates a high accuracy rate of 94% in predicting ‘non-depressed’ instances, attaining 96% precision rate and 95% recall rate. Additionally, it performs well in predicting ‘depressed’ instances, achieving an 88% precision and 89% recall rate.

The variability of depression symptoms across different age groups is examined, highlighting the risk of depression within these age cohorts. These findings are crucial for developing targeted strategies to prevent and intervene in cases of depression. Moreover, the findings aid in the creation of a reliable depression detection model capable of accurately identifying depression across a wide range of age groups. The model’s ability to precisely predict the probability of depression by considering specific attributes within each age bracket holds substantial importance. Ultimately, this promotes better mental health outcomes for individuals of all ages.

Moreover, by employing the Apriori algorithm, this study unveils robust associations between specific factors and depression within distinct age groups. By leveraging data-driven analysis, distinct patterns have emerged, showcasing the nuanced dynamics influencing mental health outcomes. For instance, in the (10–11) age group, environmental factors, family dynamics, and sleeping problems are identified as significant contributors. Moving to the (12–13) age group, parental factors, school problems, and the physical inactivity of the child strongly contribute to depression. The exploration of the mental well-being of 14–15-year-olds unveils the intricate complexities of depression, shedding light on the roles of family dynamics, school-related issues, and personal habits. Similarly, teenagers aged 16–17 face challenges such as bullying and financial hardship, which can contribute to depression. Additionally, family dynamics, school-related issues, and personal habits also play a significant role. This finding highlights the importance of recognizing these age-specific factors to better understand how different elements contribute to depression at various stages of development.

This study utilized a longitudinal data utilizing RF, Boruta, and the Apriori algorithm, to explore the association between early childhood behaviours and the subsequent development of depression. The findings shed light on the importance of early detection, identified significant predictors of depression, and explored the influence of various factors on depressive symptoms across distinct age cohorts. The results enhance understanding of the likelihood of depression at different ages and provide valuable insights for early intervention strategies and targeted interventions. By utilizing the combination of insights provided by the RF and Apriori algorithms and considering a comprehensive range of variables, this study enhances understanding of the multifaceted nature of depression and guide the development of effective prevention and intervention strategies.

## Supplementary Information

Below is the link to the electronic supplementary material.

Supplementary file1 (DOCX 16 KB)

## Appendix

See Figs. 4, 5, 6 and 7.
