# Supplementary material for: Insights into depression prediction, likelihood, and associations in children and adolescents: evidence from a 12-years study
Source: Health Inf Sci Syst. 2025 Feb 28;13(1):22. doi: 10.1007/s13755-025-00335-9 (PMC11871269; doi:10.1007/s13755-025-00335-9)
Supplement: Supplementary file 1 — Supplementary file1 (DOCX 16 KB) [file 13755_2025_335_MOESM1_ESM.docx]

#Algortihm for depression prediction, likelihood and associations

Import the necessary libraries:

pandas as pd

from scipy.stats import chi2_contingency

from imblearn.over_sampling import SMOTE

from boruta import BorutaPy

from sklearn.ensemble import RandomForestClassifier

from sklearn.svm import SVC

from sklearn.linear_model import LogisticRegression

from mlxtend.frequent_patterns import apriori

Data Preprocessing:

Exclude irrelevant variables and those with high missing data percentage:

relevant_variables = dataset.drop(['age', 'area_code', 'job_code', 'date_of_birth'], axis=1)

relevant_variables = relevant_variables.dropna(thresh=len(relevant_variables) * 0.3, axis=1)

Data Extraction:

Conduct chi-square test for association between categorical variables and outcome variable:

significant_variables = []

for column in relevant_variables.columns:

contingency_table = pd.crosstab(relevant_variables[column], relevant_variables['depression'])

chi2_stat, p_val, _, _ = chi2_contingency(contingency_table)

if p_val < 0.05:

significant_variables.append(column)

Variable Selection:

Retain relevant variables with low missing data for analysis:

selected_variables = relevant_variables[significant_variables]

Case Selection:

Combine selected cases from different datasets:

final_dataset = pd.concat([selected_cases_wave1, selected_cases_wave2, selected_cases_wave3, ...])

Handling Imbalanced Dataset:

Utilize SMOTE to balance classes:

X = final_dataset.drop('depression', axis=1)

y = final_dataset['depression']

smote = SMOTE()

X_resampled, y_resampled = smote.fit_resample(X, y)

Feature Selection:

Use Boruta alongside Random Forest for feature selection:

rf = RandomForestClassifier(n_estimators=100, n_jobs=-1, max_depth=5)

boruta_selector = BorutaPy(rf, n_estimators='auto', verbose=2)

boruta_selector.fit(X_resampled, y_resampled)

selected_features = X.columns[boruta_selector.support_]

Classification:

Apply supervised learning models (RF, SVM, LR):

rf_classifier = RandomForestClassifier()

svm_classifier = SVC()

lr_classifier = LogisticRegression()

rf_classifier.fit(X_resampled[selected_features], y_resampled)

svm_classifier.fit(X_resampled[selected_features], y_resampled)

lr_classifier.fit(X_resampled[selected_features], y_resampled)

Association Rule Mining:

Use Apriori algorithm for association rule mining:

frequent_itemsets = apriori(final_dataset, min_support=0.1, use_colnames=True)

For(consequent=depression_16_17)

Set min_support

#Max lenght of apriori n-grams

max_len = 3

frequent_items = apriori(dataset, use_colnames=True, min_support=min_support, max_len=max_len + 1)

rules = association_rules(frequent_items, metric='lift', min_threshold=1)

target = '{\ depression_16_17\'}'

results_ depression_16_17_rules[rules['consequents'].astype(str).str.contains(target, na=False)].sort_values(by='confidence', ascending=False)

antecedentlist[i] = {antecedent 1, antecedent 2, antecedent 3,... where lift value > 1)

while(len(antecedentlist)!=0)

{

For(consequent=antecedentlist[i])

antecedentlist.pop(0)

frequent_items = apriori(dataset, use_colnames=True,min_support=min_support, max_len=max_len + 1)

rules = association_rules(frequent_items, metric='lift', min_threshold=1)

target = '{\ consequent \'}'

results_consequent[rules['consequent'].astype(str).str.contains(target, na=False)].sort_values(by='confidence', ascending=False)

antecedentlist.append(new antecedent) (where lift value > 1)

}
